# Novel mechanistic insights underlying fungal allergic inflammation

**DOI:** 10.1371/journal.ppat.1011623

**Published:** 2023-09-13

**Authors:** Yufan Zheng, Eric V. Dang

**Affiliations:** Molecular Mycology and Immunity Section, Laboratory of Host Immunity and Microbiome, National Institute of Allergy and Infectious Diseases, National Institutes of Health, Bethesda, Maryland, United States of America; Universitat Zurich, SWITZERLAND

## Abstract

The worldwide prevalence of asthma and allergic disorders (allergic rhinitis, atopic dermatitis, food allergy) has been steadily rising in recent decades. It is now estimated that up to 20% of the global population is afflicted by an allergic disease, with increasing incidence rates in both high- and low-income countries. The World Allergy Organization estimates that the total economic burden of asthma and allergic rhinitis alone is approximately $21 billion per year. While allergic stimuli are a complex and heterogenous class of inputs including parasites, pollens, food antigens, drugs, and metals, it has become clear that fungi are major drivers of allergic disease, with estimates that fungal sensitization occurs in 20–30% of atopic individuals and up to 80% of asthma patients. Fungi are eukaryotic microorganisms that can be found throughout the world in high abundance in both indoor and outdoor environments. Understanding how and why fungi act as triggers of allergic type 2 inflammation will be crucial for combating this important health problem. In recent years, there have been significant advances in our understanding of fungi-induced type 2 immunity, however there is still much we don’t understand, including why fungi have a tendency to induce allergic reactions in the first place. Here, we will discuss how fungi trigger type 2 immune responses and posit why this response has been evolutionarily selected for induction during fungal encounter.

## Cellular response pathways in type 2 immunity

**Fig 1:**
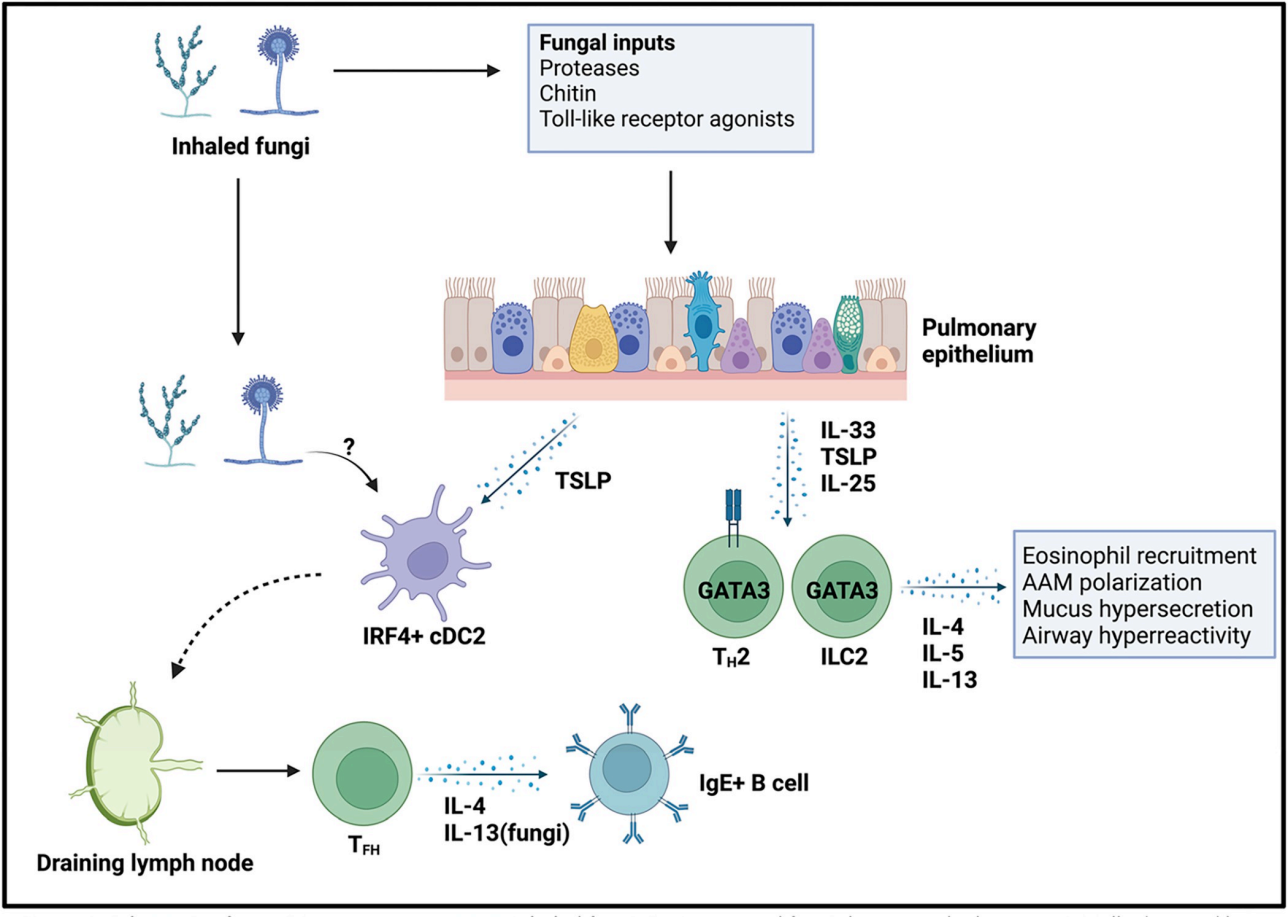
Schematic of type 2 immune responses to inhaled fungi. Environmental fungi that enter the lungs are initially detected by airway epithelial cells. In response to fungi inputs, the airway epithelium produces a trio of innate type 2 cytokines (IL-33, TSLP, IL-25) that act on tissue resident lymphocytes such as ILC2s and T_H_2s to stimulate their expression of IL-4, IL-5, and IL-13. These lymphocyte-derived cytokines act on a variety of cell types to drive the physiological and pathophysiological outputs of allergic inflammation. Epithelial-derived cytokines such as TSLP also likely act in concert with fungal molecules on dendritic cells to initiate adaptive type 2 responses. Locally activated IRF4+ cDCs migrate to the draining lymph nodes where they active naïve CD4+ T cells to become IL-4-producing T_FH_ cells that drive antibody IgE isotype switching. Created with Bio-render.

Type 2 immunity or allergic inflammation is comprised of both innate and adaptive immune arms (Fig 1) [1]. Adaptive type 2 immunity is characterized by the induction of T helper 2 (T_H_2) cells that produce a group of type 2 cytokines including interleukin-4 (IL-4), IL-5, IL13, and B cell class switch recombination towards IgE antibodies that bind to FcεR1 on mast cells and basophils resulting in anaphylactic reactions upon secondary exposure to allergen [2, 3]. Induction of T_H_2 cells requires dendritic cell (DC) capture of allergens and subsequent priming of naïve CD4^+^ T cells to drive upregulation of the GATA3 transcription factor [4]. While T_H_2 cells are described as being producers of IL-4, IL-5, and IL-13, studies using cytokine reporter mice found that T cells, in this case CXCR5+PD1+ T follicular helper cells (T_FH_), only produce IL-4 in lymph nodes in response to parasite infection, whereas they dominantly produce IL-5 and IL-13 after entering peripheral tissues such as the lungs [5]. This suggests that full T_H_2 differentiation is a multistep process that requires initial priming in the lymph node followed by confirmation signals encountered in tissues, such as IL-33 [6, 7]. On the other hand, in response to fungal challenges such as *Alternaria alternata* extract, T_FH_ cells can produce IL-13 that helps promote IgE class switch recombination in B cells [8]. Why fungal inputs seem to be special in their ability to promote IL-13 production from T_FH_ will be an important question for future studies.

The innate arm of type 2 immunity is based on a group of tissue resident innate lymphocytes, namely group 2 innate lymphoid cells (ILC2s) [9, 10]. Epithelial cells in barrier tissues initially sense allergic stimuli and then rapidly secrete cytokines that act directly on ILC2s to promote their activation [11]. Upon activation, ILC2s secrete IL-5 and IL-13, which have a variety of outputs including recruitment and activation of eosinophils, alternative activation of macrophages, differentiation of goblet cells, and smooth muscle cell contraction [12]. In some cases, the distinction between adaptive and innate type 2 inflammation becomes blurry. For example, after differentiation and recruitment of T_H_2 cells into tissues such as the lungs, a subset of these cells will persist as tissue resident memory CD4+ T cells that can be subsequently activated either by TCR engagement or via stimulation by epithelial cell-derived cytokines in a TCR-independent manner [7, 13].

### Roles for commensal and environmental fungi in allergic sensitization

While fungal sensitization is present in a large percentage of atopic individuals, it remains unclear how and when this process occurs. Studies that have longitudinally tracked fungal allergic reactivity to *Alternaria*, *Aspergillus* or *Cladosporium* by skin prick test over three decades showed that sensitization is age-dependent and has a peak in patients aged 21–40 years old [14]. Given the ubiquitous presence of fungi in the environment (it is estimated that humans breathe between 1000 and 10 billion fungal spores per day) it seems reasonable that allergic sensitization could occur *de novo* in adults. However, this does not preclude the possibility that genetic or environmental factors create conditions favorable for atopic sensitization to fungi, particularly since only around 20% of atopic individuals show fungal reactivity. For example, a large number of children with cystic fibrosis (CF) are colonized by *A*. *fumigatus*, however not all of these patients develop allergic bronchopulmonary aspergillosis (ABPA). Interestingly, pediatric CF patients with ABPA have lower serum vitamin D levels; mechanistically, vitamin D causes reduction of OX40L and increased TGF-β expression by dendritic cells, favoring conversion of T cells towards regulatory T cells as opposed to T_H_2 [15].

There is a growing body of literature suggesting that early life fungal dysbiosis during a critical developmental window may be a triggering factor for subsequent allergic reactions [16–18]. Gut microbiome sequencing studies have found enrichment of intestinal fungal species, such as *Candida* and *Rhodotorula* species in infants who subsequently have higher asthma risk when followed longitudinally [18]. In a non-industralized infant cohort from Ecuador, another group found an association between fungal dysbiosis, in particular increased abundance of the yeast *Pichia kudriavzevii* in the intestine of 3 month old babies, and subsequent increased risk of asthma at age 5 [16]. Consistent with the human data, studies in mouse models using neonatal exposure to *P*. *kudriavzevii* followed by adult challenge with house dust mite (HDM) extract showed increased allergic inflammation in response to the heterologous HDM challenge [19]. What remains unclear is which factors result in the overgrowth of these fungi in individuals with allergic predisposition. Studies in humans have suggested that early life use of antibiotics is associated with increased asthma and eczema risk in later life, although other work argued the effect is more selective to recurrant wheeze [20, 21]. It would be of interest to examine whether fungi could provide a mechanistic link between antibiotic usage and asthma sensitization, since use of these drugs can promote fungal overgrowth [22–24].

On the other hand, fungal intestinal dysbiosis in adult mice can also be a sensitizing factor for pulmonary allergen challenge outside of this neonatal window. In one study, the authors put adult mice on drinking water containing fluconazole to deplete endogenous fungi and challenged these mice with HDM. Fluconazole-containing drinking water increased the pulmonary response to HDM, which might initially argue that fungi paradoxically antagonize the allergic response [25]. However, the authors subsequently observed that fluconazole depletes drug-sensitive fungi such as *Candida* species, while enriching for three species of drug-resistant fungi, *Wallemia mellicola*, *Aspergillus amstelodami*, and *Epicoccum nigrum*. Subsequent experiments confirmed that these three species were sufficient to promote augmented HDM-response when gavaged into germ-free mice, and that fluconazole-treatment had no effect in mice colonized with defined bacterial communities but lacking fungi [26, 27].

Other work has shown that depletion of gut bacteria with antibiotics followed by gut colonization with *C*. *albicans* results in an enhanced response to HDM or *Aspergillus* spore intranasal challenge as a result of increased pulmonary ILC2 numbers or T_H_2 responses, respectively [28, 29]. An important remaining question from these studies is how signals derived from intestinal fungi alter the environment in distal organs such as the lungs? One intriguing possibility is that T cells primed in the gut are cross-reactive to inhaled allergens and then migrate to the lungs to form T_RM_ cells that expand upon subsequent pulmonary allergenic antigen exposure. While many of these studies used HDM as the pulmonary challenge, it would be interesting to test what happens with inhaled fungal extracts such as *A*. *alternata*, although cross-reactivity between fungal and HDM antigens cannot be ruled out. ILC2s have also been reported to egress from the intestine in response to alarmins and possess the ability to seed distal organs such as the lungs, which could provide a partial explanation for the *C*. *albicans* observations [30, 31].

### Sensing pathways that drive type 2 inflammation in response to fungi

While it is clear that fungal exposure is a strong inducer of type 2 allergic inflammation, the mechanisms underlying this process remain murky. The most well-studied fungal components capable of inducing type 2 inflammation are fungal proteases [32–34] and chitin [35–38]. *Aspergillus*-derived proteases have been shown to catalyze the production of fibrinogen cleavage products (FCPs), which were argued to directly bind to toll-like receptor 4 (TLR4) [39]. It has also been reported that TLR4 is required for protease-induced type 2 immune responses *in vivo* [40]. This work is consistent with previous literature showing that low-dose LPS can drive pulmonary type 2 responses and that TLR4 is required for HDM-induced allergic airway disease [41–43]. While these studies position TLR4 as part of an induction pathway for type 2 responses, there is other literature suggesting that this receptor supports an amplification pathway [44]. In a recent study, TLR4 activation by a secreted fungal effector protein was shown to promotes STAT3-dependent upregulation of IL4Ra, resulting in amplification of macrophage alternative activation in response to IL-4, consistent with previous work on Mycobacterial infection [45–48]. While further mechanistic studies are required, these data might suggest that in some contexts TLR4 can be an initiator whereas in other contexts it acts as an amplifier of allergic inflammation.

**Fig 2:**
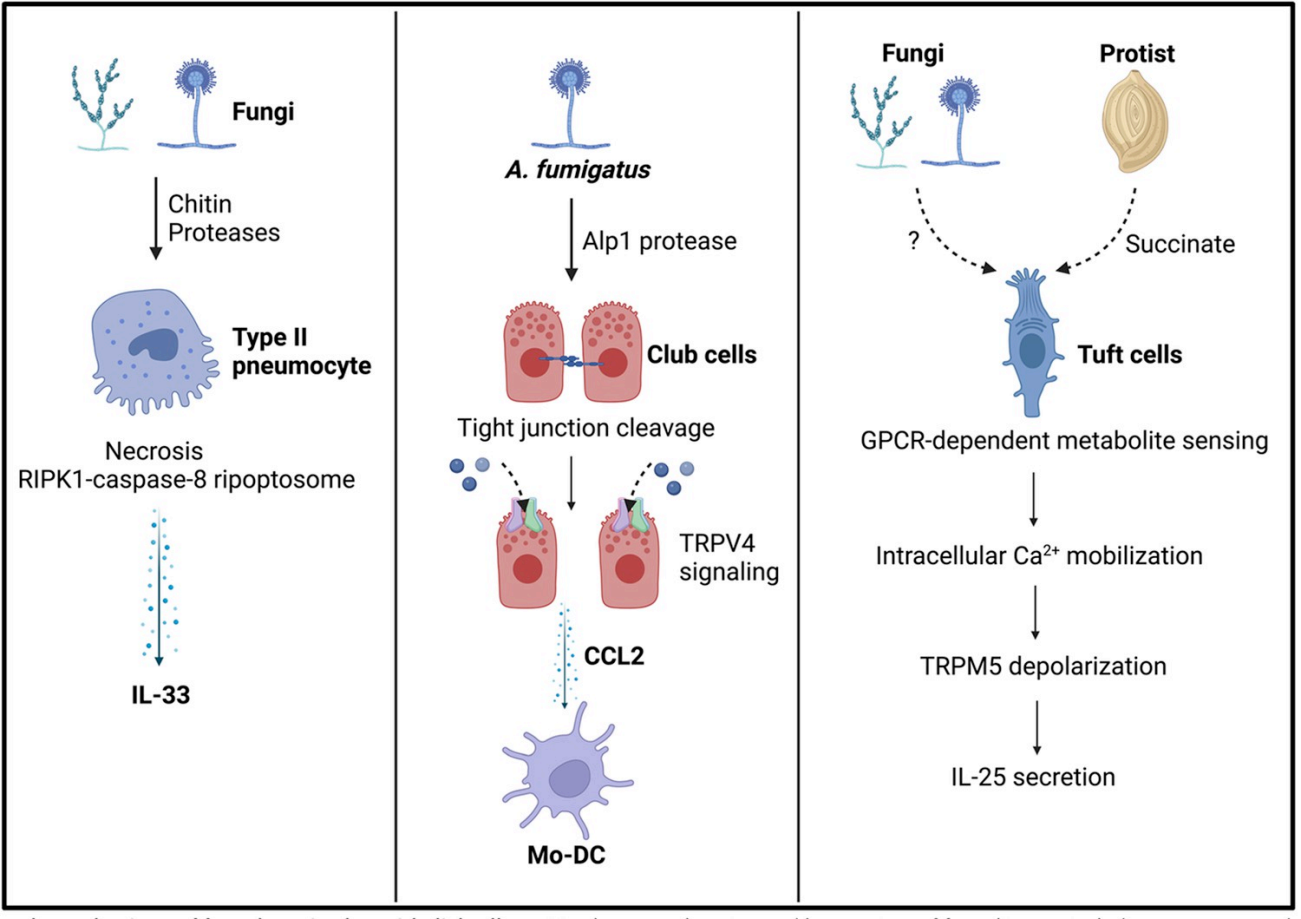
Example mechanisms of fungal sensing by epithelial cells. IL-33 release can be triggered by a variety of fungal inputs including proteases and chitin. While the exact mechanisms of input detection remain uncertain, type II pneumocytes have been identified as a dominant source of this cytokine. In response to fungal-derived damage, type II pneumocytes will activate necroptosis or ripoptosome signaling that results in secretion of the alarmin IL-33 (Left Panel). Another mechanism of damage sensing by epithelial cells involves direct recruitment of antigen-presenting cells in response to tissue disruption. In this model, the *A*. *fumigatus*-derived protease Alp1 cleaves tight junction proteins in pulmonary Club cells, resulting in TRPV4-dependent release of CCL2 in order to recruit monocyte-derived dendritic cells (Mo-DC) that are required for adaptive type 2 responses. Finally, while tuft cells are known to be activated by stimuli that are agonists for gustducin-coupled GPCRs, there are no known fungal derived molecules that directly trigger tuft cell activation. This is in contrast to models for tuft cell activation in response to intestinal protists, which is dependent on microbe succinate secretion. Created with Bio-render.

Direct epithelial cell damage induced by fungal proteases is another key pathway for initiation of type 2 inflammation. One important study showed that a gated calcium channel, transient receptor potential cation channel subfamily V member 4 (TRPV4), on the bronchiolar epithelial club cells sensed the *A. fumigatus* protease Alp1-induced injury of tights junction and drove monocyte-derived DC recruitment, which subsequently sensitized allergic inflammation [49]. Epithelial cell sensing of fungal protease-dependent damage may play an important role in the initiation stage of type 2 immunity by coupling to production of ILC2 activating cytokines such IL-33, TSLP, and IL-25, which are all reported to contribute to fungal protease-induced type 2 immunity [50]. Consistent with this notion, it has been shown that IL-33 release by pulmonary epithelial cells occurs downstream of RIPK1-caspase-8 ripoptosome activation in response to a plethora of allergic inputs, including *A*. *alternata* and *A*. *fumigatus* extracts [51, 52]. These results are consistent with the Palm/Medzhitov model for allergic inflammation, which posits that allergy exists to clear the body of noxious environmental agents that drive tissue damage, rather than being a misdirected parasite response [53]. An important research avenue for future studies will be understanding the full repertoire of damage recognition pathways that function upstream of the secretion of these innate type 2 cytokines (Fig 2).

Chitin is a key structural component of the fungal cell wall and is also the second most abundant polysaccharide in the nature. A series of seminal studies found that intranasal administration of chitin particles is sufficient to drive type 2 inflammation, suggesting that chitin may be a key fungal macromolecule that promotes allergic responses [35, 36, 54]. Additionally, chitin extracts from *Aspergillus* found in house dust from asthmatic households were capable of driving type 2 responses in mice [36]. However, it remains unclear exactly how chitin is recognized in order to drive allergic inflammation, and there are discrepant results depending on the size of the chitin particles used [55, 56]. So far, four receptors, TLR2, NOD2, FIBCD1 and LYSMD3, are reported to recognize chitin [57–60]. However, there is a little to no experimental evidence that these molecules act as chitin recognition receptors for the initiation of type 2 immunity. As reported, chitin can induce a type 2 immune response in MYD88-deficient mice, suggesting this induction is independent from TLR signaling [35]. In *Cryptococcus neoformans* infection models, which drive strong pulmonary type 2 responses, cell wall chitin content correlated with stronger type 2 inflammation [61]. Lung-resident CD11b^+^ IRF4-dependent conventional dendritic cells were required to present antigen for T_H_2 induction in this context. Although these cells likely do not sense chitin fragments directly, they express the TSLP receptor, which may sense TSLP derived from pulmonary epithelial cells to mediate T_H_2 induction. Here again, lung epithelial cells may act as initial sensors for type 2 inputs such as chitin which then send signals to DCs to direct the T cell output.

It is also possible that no designated chitin receptor exists, rather chitin accumulation causes tissue damage that acts as a trigger of type 2 inflammation. There are two types of chitinases encoded by mammals, acidic mammalian chitinase (AMCase) and chitotriosidase (Chit1) [37]. AMCase deficiency leads to age-dependent pulmonary chitin accumulation and causes increased ILC2 responses and pulmonary fibrosis, whereas transgenic AMCase overexpression results in resistance to chitin-dependent type 2 inflammation, demonstrating that degradation of insoluble polysaccharide chitin by AMCase is protective [35, 62]. In this context, AMCase seems to be a negative regulator in type 2 inflammation, or alternatively and perhaps more likely, it sets a threshold for lung chitin levels or size beyond which they stimulate type 2 responses [63].

Although the mRNA and protein levels of *Chit1*/CHIT1 are almost undetectable in lungs of uninfected mice, this gene was found to be required for T_H_2 differentiation in *Cryptococcus* infection models [61]. In mice with cryptococcosis, T_H_2 cells are decreased by *Chit1* deletion, whereas the mice with AMCase deletion spontaneously develop a significant increase in ILC2 cells. These observations suggest that the mechanisms by which chitin induces a type 2 immune response may not be the same in different conditions, worthy of further studies. This also raises of the question of whether CHIT1 produces specialized chitin fragments that are sensed by other receptor pathways. Indeed, while the literature is inconsistent with a role for TLRs in promoting chitin-dependent allergic inflammation, there is evidence that there are clear size requirements for chitin oligomers to activate receptors such as TLR2 [57].

In the classical model, pattern recognition receptors (PRRs) expressed on DCs sense pathogen-associated molecular patterns (PAMPs) to induce cytokines that direct differentiation into T helper 1 (T_H_1) or T helper 17 (T_H_17) cells [64]. For example, C-type lectin receptors (CLRs) recognize sugar moieties such as β-1,3-glucans and α-mannans that are found on the fungal cell wall. Upon ligand binding, CLRs direct the differentiation of T_H_17 responses to promote anti-fungal immune responses [65]. As has been alluded to in this review, T_H_2 responses seem to obey different rules as there are no known DC-expressed PRRs that exclusively drive T_H_2 differentiation in response to antigen [6]. In the context of house dust mite (HDM) allergy, Dectin-1 and Dectin-2 have been reported to participate in T_H_2 differentiation [66–69]. However, it is not clear whether Dectin ligation drives production of instructional molecules for T_H_2 differentiation by DCs, or if it represents an activation signal that promotes DC migration from the lungs into the draining lymph nodes. Understanding the contributions of different PRRs on DCs in the context of fungal-induced type 2 immunity will be an important topic for future research.

### Epithelial cell-derived cytokines as initiators of fungal type 2 inflammation

Upon sensing fungi, epithelial cells in barrier tissues produce mediators that rapidly activate ILC2s or tissue resident T_H_2s. IL-33 is the best studied of these mediators, and has been shown to activate ILC2s through binding to its receptor, suppression of tumorigenicity 2 (ST2; also called IL1RL1) [70]. Recent work revealed that intratumoral fungal mycobiome-driven IL-33 contributed to the pathogenesis of pancreatic ductal adenocarcinoma (PDAC) through induction of type 2 immunity [71]. Anti-fungal treatment in mice with PDAC led to a decrease in T_H_2 and ILC2 tumor infiltration as well as an increase in survival. Previous studies in mouse models have showed that IL-33 is required for induction of type 2 inflammation caused by a variety of allergic fungi [50, 72]. For example, in *Alternaria*-challenged mice, data show that deletion of ST2 decreases the type 2 immune response with complete elimination of pulmonary eosinophilia [73, 74]. ST2-deficient mice also exhibited a better survival and lower fungal burden with decreased early production of IL-5 and IL-13 in invasive cryptococcosis [75–77]. These observations have also been linked to humans, where it was found that steroid-resistant pediatric asthma patients with fungal sensitization showed higher levels of airway IL-33 [78]. It was additionally found that human sinonasal epithelial cells produce IL-33 in response to *A. fumigatus* though the PAR2 receptor [79].

Less work has been done on the role of TSLP in the context of fungal-induced allergic inflammation. One study found that TSLPR-deficient mice have decreased production of IL-5 and IL-13 from ILC2s and diminished airway eosinophilia after challenge with *A*. *alternata* extract [80]. Consistent with these results, another group found that cultured human nasal polyp fibroblasts produced TSLP in response to stimulation with *A*. *fumigatus* or *A*. *alternata* in a TLR2-dependent manner [81]. Another group found that *A*. *alternata* extract could stimulate TSLP production in human bronchiolar epithelial cells via PAR2 activation [82]. Further work is needed to dissect the precise role(s) of TSLP in the initiation of fungal allergic inflammation and to determine the identity of the sensor pathways that drive its production *in vivo*.

More recently, IL-25 has been identified as an epithelial cell-derived cytokine that drives ILC2 activation through binding to its receptor, IL-17RB [83–86]. Interesting, through the use of fluorescent reporter mice, it was found that IL-25 is produced by a specialized group of secretory epithelial cells known as tuft cells [87–89]. Subsequently, tuft cells were found produce IL-25 and leukotriene C4, a lipid mediator, to synergistically mediate *Alternaria*-induced type 2 immunity [90]. In a *C*. *neoformans* pulmonary infection model, IL-25-deficient mice showed decreased fungal dissemination from lung to brain, although a direct contribution of tufts cells to this phenotype was not assessed [91]. In this study, authors highlighted the role of T_H_2 cells in IL-25 signaling, but a group of ILC2-like cells also has high expression of IL-17RB during cryptococcosis. These data suggest that the role of ILC2s in mediating IL-25-induced type 2 immunity in cryptococcosis and other inhaled allergenic fungi is worthy of further study [92].

While it is clear that tuft cells are important contributors to the initiation of type 2 inflammation, how these cells recognize allergic stimuli is only starting to be elucidated (Fig 2). Tuft cells are characterized by the expression of a G-protein isoform called gustducin [93]. When activated downstream of G-protein coupled receptors (GPCRs), gustducin drives release of intracellular Ca2+ stores. Intracellular Ca2+ then promotes tuft cell activation via the depolarization of transient receptor potential cation channel subfamily M member 5 (Trpm5) [94–96]. Based on this model of tuft cell activation, allergic stimuli are sensed by GPCRs. Consistent with this idea, intial studies on the mechanisms of tuft cell activation have shown that intestinal protists secrete the metabolite succinate, which is detected by SUCNR1 on tuft cells to drive IL-25 secretion and ILC2 activation [97–99]. Another study found that the taste receptor TAS1R3 plays a role in the tuft cell response to the helminth *Heligmosomoides polygyrus* [100]. While there is mounting evidence that fungi are capable of driving tuft cell activation, the identity of fungal-derived molecules that activate tuft cell expressed GPCRs remains a black box. Another possible model is that fungal-derived molecules, such as proteases, drive tissue damage in neighboring cells, which subsequently release tuft cell GPCR-activating DAMPs as has been suggested for IL-33 release [101, 102].

### Effector outputs from fungal-induced type 2 inflammation

While it is clear that fungi are strong inducers of type 2 inflammation, what is less obvious is why this mode of immune response occurs in response to this class of microbe. It is generally argued that type 2 immunity is detrimental to the host in the context of fungal infections such as *C*. *neoformans*, *H*. *capsulatum*, *C*. *immitis*, and *A*. *fumigatus* [72, 103]. Most of these arguments converge upon macrophages, which play important roles in clearance of pathogens. In response to type 2 cytokines, macrophages polarize into alternative activated macrophages (AAMs) characterized by expression of PDL2, ARG1, YM1, YM2, FIZZ1, and MRC1, among others [104]. While *in vivo* this framework is likely oversimplistic, the concept of AAM stands in contrast to classically activated macrophages (CAMs), which are activated by type 1 cytokines and display enhanced pathogen killing capacity [105, 106]. AAMs contribute to the control of inflammatory responses and play important roles in wound healing and tissue homeostasis. However, AAMs play a detrimental role in the case of many invasive fungal infections. For instance, global deletion of *Il4ra* or *Stat6* led to decreased fungal burden in lung tissues of mice infected with *C*. *neoformans* [45, 107, 108]. Mice with macrophage-specific *Il4ra* deletion also had a better survival, indicating that alternative activation weakens the ability of macrophages to defend against fungi [107]. Similar results have been found with other fungal infections [109].

While deletion of type 2 cytokine signaling in macrophages demonstrates an important role for AAMs in fungal immunopathogenesis, it remains unclear exactly why these cells, and type 2 immunity in general, are detrimental during certain fungal infections. The classic argument is that since AAMs have decreased expression of intracellular killing molecules (iNOS, etc), they may represent an intracellular replication niche. Alternate, though non-mutually exclusive, models are that AAMs directly support fungal growth via metabolite secretion or that they shape a local immunosuppressive environment *in trans* via expression of lymphocyte inhibitory molecules or recruitment of granulocytes such as eosinophils, which can antagonize protective T_H_1 responses to fungi such as *C*. *neoformans* [110–112]. Additionally, there are likely many macrophage-independent outputs of type 2 inflammation that counteract protective responses, such as mast cell activation and recruitment of eosinophils via GPR35 or direct inhibition of T cell IFNγ production by type 2 cytokines [110, 113].

Intriguingly, this concept of detrimental type 2 immunity has been exploited for therapeutic benefit in a case report of disseminated Coccidiomycosis in a previously healthy child that was non-responsive to antifungal treatment [114]. This patient was treated with subcuteneous recombinant interferon-γ, which slowed fungal disease progression but did not result in full resolution. Subsequent treatment with Dipilumab (IL-4Rα blocking antibody) resulted in complete resolution of the infection. While this was only one patient, this case report demonstrates that modulation of type 2 immune responses may open up exciting new avenues for antifungal therapy, and additionally provides evidence that this circuit plays a role in human infection in addition to mice.

**Fig 3:**
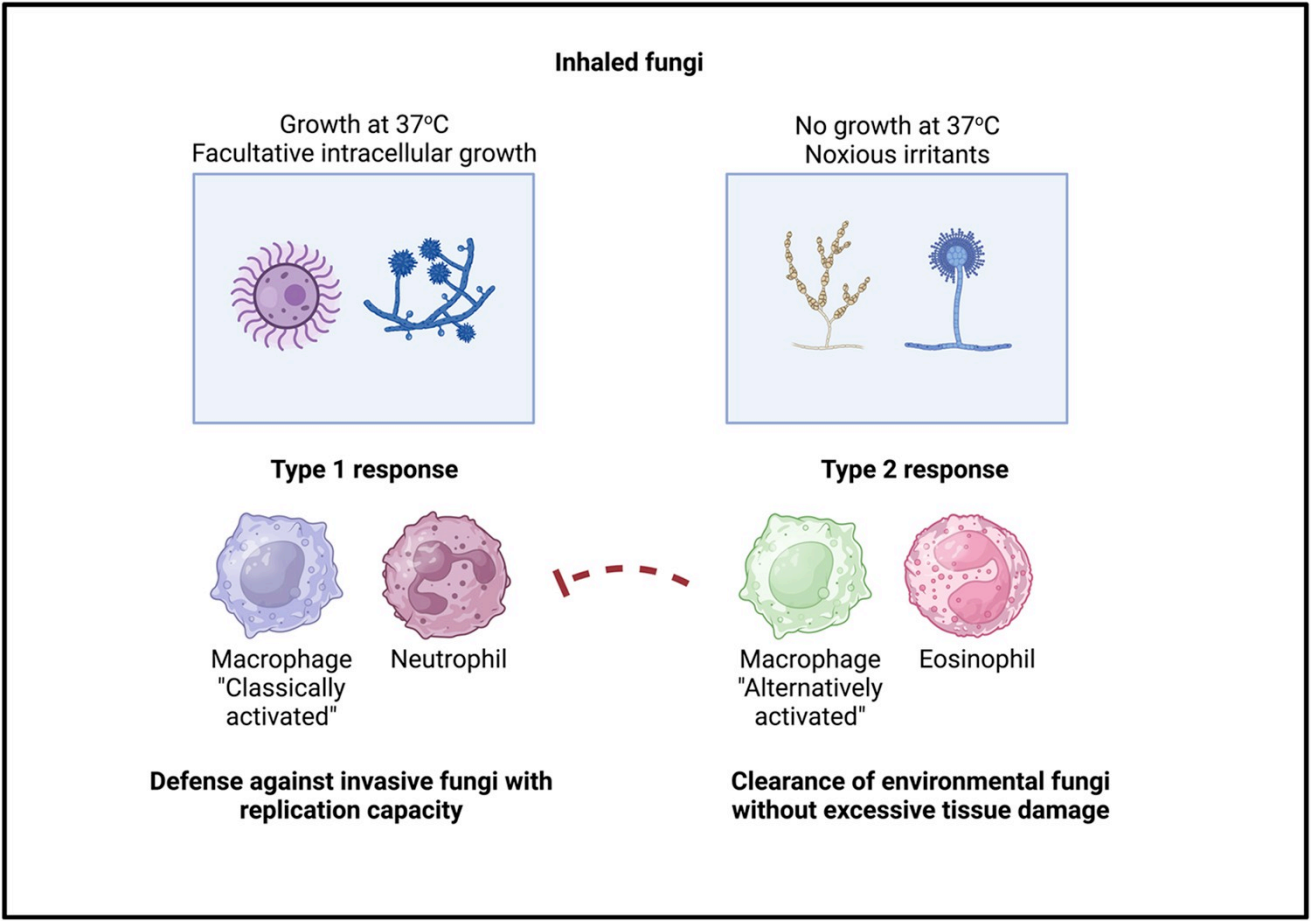
Theoretical rationale underlying type 2 immune responses to fungi. Fungi are very common drivers of allergic inflammation in mammals, which could be a result of misdirected anti-parasite responses or an evolutionarily selected response. While many studies show that type 2 immune responses are detrimental to the clearance of invasive fungal pathogens, the species that drive such infections have only recently become major sources of human infection due to the rise of HIV and increasing use of immunosuppressive drugs. One hypothesis is that type 2 responses were selected to occur in response to environmental fungi that act as inhaled irritants, but that cannot replicate at human body temperature. In this case, type 1 inflammation might pose the risk of excessive tissue damage; on the other hand, type 1 responses would be beneficial in the case of fungal that are capable of causing invasive disease. Type 2 cytokine-dependent upregulation of C-type lectin receptors such as Dectin-1 and mannose receptor in macrophages, and recruitment of innate type 2 immune cells such as eosinophils, may allow for “silent clearance” of inhaled fungi while limiting immunopathology. Created with Bio-render.

Why then induce a response that is detrimental to the host? One possibility is that the initiation of type 2 immunity in response to fungi is a misdirected anti-parasite response. Another hypothesis is that the species that cause opportunistic fungal infections are a tiny fraction of the overall fungal biodiversity encountered by humans. Invasive fungi have only become major human health problems in recent history on an evolutionary time scale due to the rise of HIV, cancer chemotherapeutics, and immunosuppressant drugs over the last three decades [115]. Additionally, the species that cause invasive human infections have the ability to grow at mammalian body temperature (37°C), whereas the majority of environmental fungi cannot. Perhaps then type 2 inflammation plays a positive role in the host response to other types of fungi or fungal-derived molecules encountered by mammals on a more regular basis (Fig 3). One intriguing point on this front is the long-held observation that type 2 cytokines strongly upregulate fungal recognition receptors such as Dectin-1 and mannose receptor (MRC1) in macrophages [116, 117].

A tempting hypothesis is that AAMs function to clear non-replicating environmental fungi from barrier tissues via increased phagocytic capability, but without driving a hyper-inflammatory response that would be overkill for this class of microbe. There is some data that hints at this: in one study it was found that IL-33 promotes the alternative activation of pulmonary macrophages in order to drive enhanced killing and clearance of *Pneumocystis murina* [118]. Understanding differential responses to non-replicating environmental fungi versus facultative intracellular yeasts may also provide insights underlying the paradox that many fungi are capable of inducing type 2 immune responses, whereas only a limited number of species overtly sensitize and exacerbate allergic asthma. An important broader question on this front is whether there are specialized inputs or processes that differentiate induction of type 2 immunity in response to specific fungi that promote allergic asthma? Alternatively, perhaps the inputs are universal but different unidentified downstream factors control the output of type 2 inflammation.

Interestingly, eosinophils have also been shown to possess fungal killing capability. Eosinophils can bind fungi via CD11b integrin and then release cytotoxic granules that directly kill *A*. *alternata*, an environmental mold that is a major driver of allergic airway disease [119]. Similar results were found after incubation of human eosinophils with extracts derived from environmental fungi such as *A*. *alternata*, *A*. *versicolor*, *C*. *albicans*, *C*. *herbarum*, *C*. *spicifera*, and *P*. *notatum* [120].

Lastly, a recent study found that intranasal challenge of mice with *C*. *albicans* results in a pulmonary type 2 response trigger by secretion of the peptide toxin candidalysin from the yeast that is sensed by platelets [121]. Interestingly, the authors found that activation of type 2 responses by this sensing pathway was protective against pulmonary *Candida* infection.

### Questions for future research

A better understanding of type 2 immunity in response to fungi is essential for the development of clinical therapeutic strategy for patients suffering from allergic diseases. We would like to raise some key questions for the future study:

1. How universal is damage sensing in the initiation of fungi-induced type 2 immunity? Which cell types are the direct sensors of these damage signals? A study in bacterial infection reveals that Nav1.8+ nociceptors, sensory receptors for painful stimuli, directly sense bacteria to modulate immune responses, indicating a critical role of the nervous system in sensing microbes [122]. Whether neurons sense tissue damage induced by fungi to initiate the type 2 immunity is also worthy of further study. Additionally, understanding the downstream signal transduction mechanisms from these potential sensors will be a fruitful avenue for future studies.
2. What role do tuft cells play in the initiation of type 2 inflammation in response to fungi? Are they activated by damage signals or do they recognize fungal-specific molecules that trigger IL-25 production?
3. Why do relatively few species of fungi (*Alternaria*, *Aspergillus*, *Cladosporium*, *Penicillium*) account for the majority of allergens that sensitize and exacerbate allergic asthma? Is this simply a feature of environmental exposure (i.e. these happen to be the fungal species that grow in modern indoor environments), or are there unappreciated features of these species that specifically drive allergic sensitization? On the flip side, how/why do species such as *C. neoformans* induce type 2 immune responses without promoting overt asthma phenotypes?
4. How does intestinal fungal dysbiosis drive increased allergic inflammatory responses in distal organs? Does this involve cell migration from the intestines to the lungs that then promote enhanced responses to heterologous allergic stimuli? Another possibility is that intestinal fungi such as *W*. *mellicola*, P. *kudriavzevii*, *A*. *amstelodami*, and *E*. *nigrum* secrete metabolites that enter the circulation and tune inflammatory responses at distal organs.
5. Why are type 2 responses turned on in response to fungi? Is this a misdirected parasite response that is detrimental in the case of invasive fungal infections due to an unfortunate accident? Or is allergic inflammation an evolutionarily selected pathway that helps barrier tissues clear certain classes of environmental fungi?
